# Endovascular stenting for extracranial carotid artery aneurysms: Experiences and mid-term results

**DOI:** 10.1097/MD.0000000000005442

**Published:** 2016-11-18

**Authors:** Leng Ni, Zuo Pu, Rong Zeng, Rui Zhang, Yue-hong Zheng, Wei Ye, Chang-Wei Liu

**Affiliations:** Department of Vascular Surgery, Peking Union Medical College Hospital, Peking Union Medical College & Chinese Academy of Medical Sciences, Beijing, China.

**Keywords:** Bare stent, covered stent, endovascular, extracranial carotid artery aneurysm, stenting

## Abstract

The aim of this study was to investigate the safety and effectiveness of endovascular stenting for extracranial carotid artery aneurysms (ECAAs) and evaluate the mid-term outcomes.

Twelve consecutive symptomatic patients (mean age 43.8 ± 14.9 years; 8 men) with ECAAs who were treated with endovascular stenting between 1997 and 2015 were retrospectively analyzed. Clinical follow-up data including symptoms and neurological events were obtained from outpatient records. Imaging follow-up with duplex ultrasound and/or computed tomographic angiography (CTA) was performed to examine the aneurysm obliteration and patency of the stents at 3, 6, 12 months and yearly thereafter.

A total of 5 true aneurysms and 7 pseudoaneurysms were included in our series. Neurological symptoms (n = 5, 41.7%) and a pulsatile neck mass (n = 5, 41.7%) were the most common presenting symptoms. Endovascular stenting procedures were technically successful in all cases; 3 patients received bare stents, and 9 patients received covered stents. No perioperative neurologic or cardiopulmonary complications occurred. Over a period of follow-ups (mean 21.8 ± 25.1 months), all patients were alive and free from neurological or other adverse events. All aneurysms were completely excluded except for 1 patient who was exposed to a residual medium leaking into the aneurysm sac. No reintervention was performed in this specific patient because aneurysm growth or significant clinical symptoms did not occur. Recurrent restenosis assessed by CTA imaging at 12 months occurred in 1 (8.3%) patient in our series. Target lesion revascularization for this hemodynamic restenosis was treated with placement of an additional stent.

In our series, endovascular stenting for ECAAs was found to be safe, effective, and proved to have promising mid-term results. Although long-term results need to be further explored, advantages including less procedure-related complications and a shorter recovery time make endovascular stenting an attractive option for ECAAs, especially for the patients who are unfit for traditional open surgery.

## 1 Introduction

Extracranial carotid artery aneurysm (ECAA) is a rare peripheral arterial aneurysm, accounting for less than 1% of all arterial aneurysms.^[1]^ According to the meta-analysis description, most of the ECAAs were pseudoaneurysms, whereas true aneurysms took up <10% of ECAAs.^[2]^ The etiology of ECAAs is diverse and mainly includes atherosclerosis, infection, fibromuscular dysplasia, connective tissue disease, and trauma or spontaneous dissection.^[3,4]^ Most ECAAs present as a pulsatile cervical mass or a cranial nerve dysfunction resulting from local compression. However, as a potential source of emboli to the brain, ∼50% of patients suffered from transient ischemia attacks (TIA) or minor strokes before being diagnosed with ECAA.^[5–7,13]^

Previous studies reported a stroke prevalence of 50% and a mortality of 60% to 70% when ECAAs are left untreated.^[8]^ Conservative medical treatments including anticoagulation or antiplatelet drugs have been reported to lower the risk of ischemic events but are not effective at resolving lesions; thus, a long-term risk of embolization is possible.^[9,10]^ Since Dimtza^[11]^ performed the first successful carotid aneurysm excision in 1952, aneurysm resection with or without carotid artery reconstruction is regarded as the first-line option for surgical treatment. Currently, with the increasing availability of endovascular surgery, endovascular intervention, as an alternative, outshines open surgery in highly located aneurysms or hostile neck conditions caused by interventions such as radiation therapy and previous neck surgery. Research of endovascular treatment of ECCAs in large scale is rare. Therefore, we share our center's experience during the past 18 years and discuss the mid-term outcomes of endovascular treatment with ECAAs.

## 2 Methods

### 2.1 Patients

Between January 1997 and December 2015, the medical records of patients presenting with ECAA, defined as a dilatation >50% of the diameter compared to the expected healthy carotid artery,^[12]^ were reviewed. Fifty-four symptomatic ECAAs were identified in 50 patients, 4 of who had bilateral occurrences. Through clinical evaluation, all these patients who had defined indication for surgical treatment were consecutively admitted to our hospital. The choice of treatment strategy depends on 3 main factors including patients’ general conditions, aneurysm location, and patient preferences. Among these 50 patients, 13 patients with 16 aneurysms were given conservative treatment, 25 patients received surgical treatment, and 12 patients received endovascular intervention.

Demographic characters and medical data were recorded for each ECAA patient who received endovascular therapy (Table 1). Our data set includes age, gender, clinical presentation, ECAA etiology, aneurysm type according to the classification of Attigah et al,^[13]^ size and shape of the ECAA, endovascular procedures, information regarding stenting, postoperative complications and antiplatelet or anticoagulant therapy. Informed written consent was obtained from each patient after admitted to our hospital. The Institutional Review Board at Peking Union Medical College Hospital approved all study procedures.

**Table 1:**
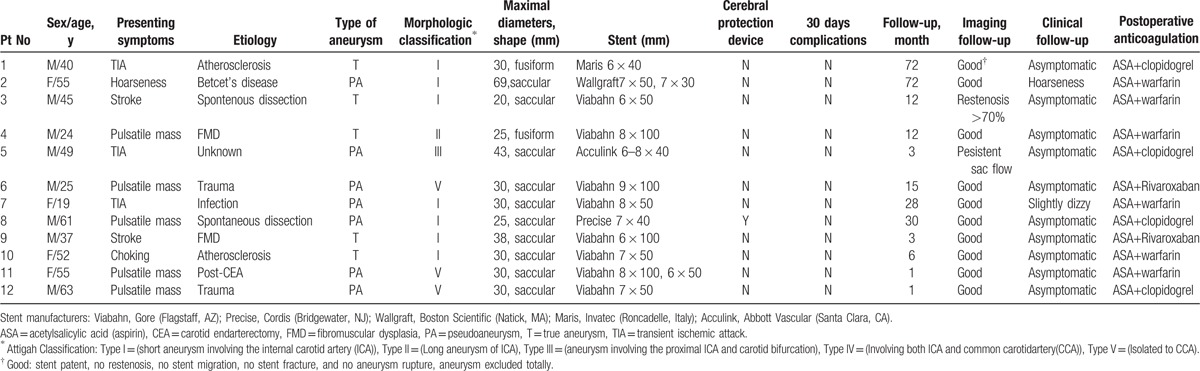
Patients demographic, endovascular treatment, and follow-up.

### 2.2 Endovascular procedures

**Figure 1:**
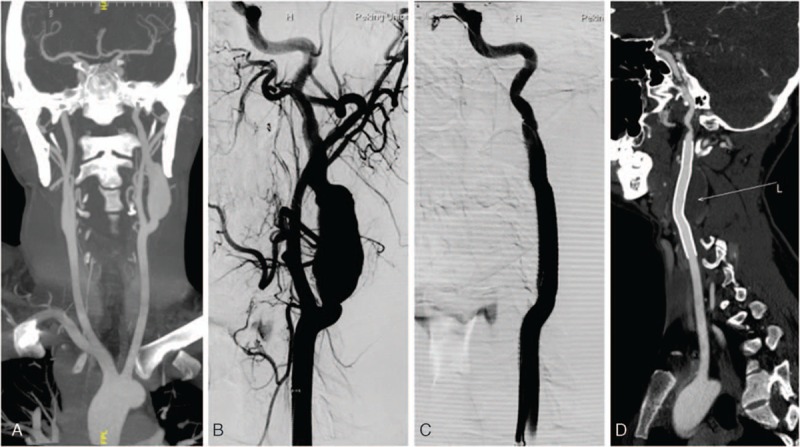
A 24-year-old male presented with a pulsatile mass on his left neck. Preoperative CTA indicated a true aneurysm in the left internal carotid artery (ICA) near the skull base, which is classified as Attigah type II (A). A guide catheter was placed in the ostium of the common carotid artery (CCA). After angiography was carried out through a guide catheter (B), an 8 × 100 mm covered stent (Viabahn, W.L. Gore) was deployed in the segment of the lesion. Immediate angiography showed absolute obliteration of the aneurysm sac with no endoleak (C). Follow-up CTA imaging results at 12 months showed stent patency without restenosis or endoleak (D). CCA = common carotid artery, CTA = computed tomographic angiography, ICA = internal carotid artery.

**Figure 2:**
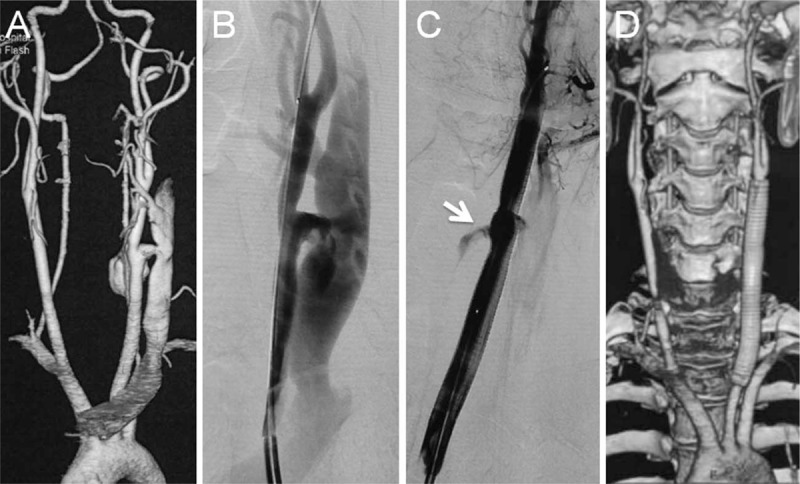
A 25-year-old male who suffered gunshot trauma to the left neck 1 month prior to admission presented with progressive swelling on the left side of his neck. Preoperative CTA showed a large pseudoaneurysm of the left CCA combined with an arteriovenous fistula (AVF) (A). After anatomic details of the lesion were confirmed with angiography (B), a 9 × 100 mm covered stent (Viabahn, W.L. Gore) was deployed to cover the lesion. Immediate angiography indicated a slight contrast leakage into the aneurysm sac (white arrows in C), but the AVF was completely excluded (C). Follow-up CTA at + /15 months indicated stent patency with complete exclusion of the aneurysm sac and AVF. AVF = arteriovenous fistula, CCA = common carotid artery, CTA = computed tomographic angiography.

Endovascular procedures were performed on 11 patients under local anesthesia with continuous hemodynamic monitoring according to the standard hospital protocol for carotid artery stenting procedures. Only 1 patient (Patient No. 9) had the procedure performed under general anesthesia. The internal carotid artery (ICA) access was obtained in these 11 patients through the femoral artery approach. After femoral artery access was obtained, heparin was administered according to the standard protocol (100 U/kg). A guide catheter through the support of a 0.035-inch stiff guidewire was placed in the ostium of the common carotid artery (CCA) with fluoroscopic visualization and applicable roadmap guidance if necessary. After carefully maneuvering the soft guidewire past the lesion, a covered stent or self-expanding bare stent was delivered to a proper position under the guidance of roadmap. The diameter of stent could be the same as or bigger than that of the normal artery but no more than 1 mm. Nondiseased carotid artery with a length of ∼1 cm just proximal and distal to the aneurysm was used for the landing zones of stent grafts. After the stents were deployed to cover the aneurysm, multiple control angiographies were obtained to confirm the exclusion of the aneurysm sac from the circulation. If apparent endoleak was present in the aneurysm sac, postdeployment balloon dilatation was performed in the proximal and distal ends of the graft to better appose it to the vessel wall for the full exclusion of the aneurysm. (Examples of endovascular treatment are shown in Figs. 1 and 2.) According to our experience that the risk of distal embolism is relatively low, so cerebral protection devices were selectively applied to 1 patient (Patient No. 8).

A hybrid operation was performed in Patient No. 9 with a highly located and surgically inaccessible ECAA. The proximal ICA in this patient was severely kinked, so we did not believe a covered stent could be safely advanced to the area of concern. The following steps were the major steps of the hybrid operation. The carotid artery was surgically exposed and controlled through a classical laterocervical approach. Following heparin administration and clamping of the CCA, ECA, and ICA, respectively, the kinked ICA was corrected after it was divided at the carotid bifurcation. The proximal portion of ICA was ligated, and the distal portion was anastomosed end-to-end to a prosthetic graft (5 mm–10 cm, Gore) to extend the working length for stent deployment. The prosthetic graft was punctured after clipping the free end, and then a 7F short sheath was inserted into the prosthetic graft obtain access for the guidewire. After a covered stent was deployed to cover the aneurysm, the redundant segment of prosthetic graft was moved, and an end-to-side anastomosis was performed between the rest of the prosthetic graft and the CCA.

### 2.3 Follow-up

Follow-up data consists of clinical and imaging information. Clinical information was obtained from outpatient clinic records, which included late mortality, reintervention, late surgical conversion, major/minor stroke or TIA, symptom improvement and any complications after treatment. Imaging information include CTA and duplex ultrasound imaging of the carotid artery performed at 3, 6, and 12 months after the procedure and annually thereafter to assess the following outcomes: stent-graft migration, stent-graft fracture, aneurysm rupture, thrombosis of aneurysm sac, endoleak, stent-graft patency. All patients continued to receive lifelong aspirin. The patients who underwent self-expanding bare stent placement were given 75 mg clopidogrel for 6 months after the procedure. For the patients who received the covered stenting, regular anticoagulation treatment (warfarin or rivaroxaban) was added for at least 6 months.

## 3 Results

Twelve patients with ECAA successfully received endovascular stenting. The mean age was 44 years old (range: 19–63 years old) with a slight preponderance of men (8 males and 4 females). Judged by the imaging characteristics, 5 ECAAs were true aneurysms, and the other 7 ECAAs were pseudoaneurysms. According to the classification of Attigah, 7 lesions were type I, 3 lesions were type V, 1 lesion was type II and 1 lesion was type III. The average diameter was 3.4 cm (range: 2.0–6.9 cm). Five lesions were accompanied by the presence of thrombus in the aneurysmal sac. The primary symptoms experienced by the patients in our series included neurologic ischemia (TIA in 3 patients and strokes in 2 patients), a pulsatile mass in 5 patients, and local compression in 2 patients that caused hoarseness and choking when drinking water. Etiology was atherosclerosis in 2 patients, fibromuscular dysplasia in 2 patients, spontaneous dissection in 2 patients, neck trauma in 2 patients, Behcet's disease in 1 patient, carotid endarterectomy in 1 patient, infection in 1 patient, and unknown etiology in 1 patient.

One patient underwent hybrid surgery because of severely kinked ICA, and the other 11 patients received endovascular stenting alone. Fourteen stents were deployed at the predetermined position in the 12 patients. Three self-expanding bare stents were deployed in 3 cases, and 11 covered stents were deployed in the remaining 9 patients. Apart from 1 patient (Patient No. 8), the other 11 patients did not use cerebral protection devices during the procedures. According to the results of immediate angiographic imaging after the procedure, the aneurysms of 11 patients implanted with covered stents were absolutely obliterated. In contrast, the filling of contrast medium into the aneurysm sac was decreased in 3 patients who received bare stents. No deaths or major complications, such as strokes, occurred during the operation or within 30 days of intervention in this series.

**Figure 3:**
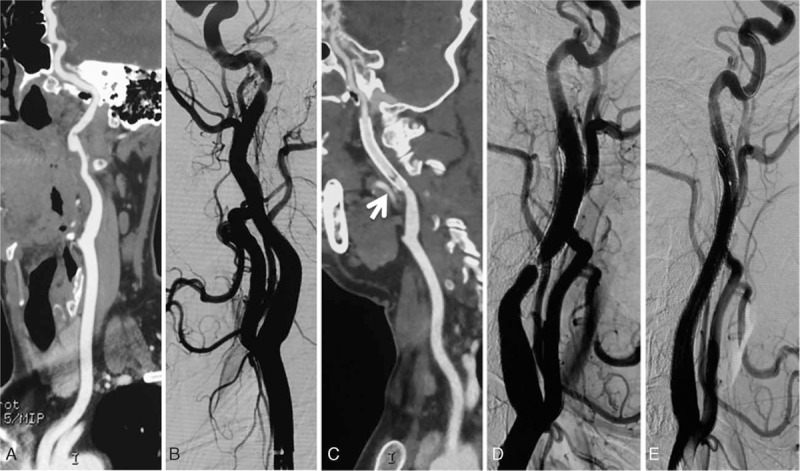
A 45-year-old male suffered from a minor stroke before being diagnosed with a dissecting aneurysm in a high location of the left ICA through CTA examination (A). Endovascular treatment was carried out with deployment of a 6 × 50 mm covered stent (Viabahn, W.L. Gore)(B). Follow-up CTA imaging at 12 months indicated that the level of in-stent restenosis was >70% (marked by white arrows in C). After angiographic assessment (D), an additional stent was placed in the segment of the restenotic lesion after balloon angioplasty with a cerebral protection device in place (E). CTA = computed tomographic angiography, ICA = internal carotid artery.

The median duration of follow-up was 22 months (range: 3–72 months). All patients were alive, and no one received any reintervention or surgical conversion. No stroke or TIA occurred during the follow-up period. Ten patients (83.3%) were asymptomatic; however, 1 patient (Patient No. 2) experienced no change in hoarseness from the preoperative status, and another patient (Patient No. 7) experienced slight dizziness. On the basis of the imaging information, no stent migration, stent fracture, endoleak, and aneurysm rupture were identified in this series. However, the rate of aneurysm sac thrombosis is 91.7% (11/12), except for 1 patient (Patient No. 5) with a small amount of contrast leaking into the aneurysm sac at 3 months. We chose an observation strategy for the patient because of a lack of aneurysm growth and no significant clinical symptoms. All stents were patent except for the covered stent in 1 patient (Patient No. 3), who developed hemodynamic restenosis 12 months after the procedure. An additional bare stent was placed in the segment of the restenosis after balloon angioplasty under the security of cerebral protection device (Fig. 3).

## 4 Discussion

As ECAA is a rare vascular disease, there exist a lack of adequate experience and guidelines in terms of choices of treatment and treatment optimization. Conservative management is associated with a 71% mortality rate.^[14]^ In the past 5 decades, the surgical technique of aneurysm resection combined with reconstruction of the carotid arterial system has been regarded as the golden standard.^[6,9,15,16]^ However, postoperative complications are more frequent in surgical procedures because of their greater invasiveness. Cranial nerve injuries, which is the most common complications, range from 11% to 26%.^[17–19]^ Moreover, when the aneurysm is located near the skull base or the patients have a hostile neck from a previous neck surgery or radiation, open surgery is challenging for exposing the distal ICA. Recently, advanced endovascular therapy has become more attractive with less invasiveness. Additionally, this therapy can be applied to areas that are difficult to access under open surgery and bears a lower risk of cranial nerve injuries. Li et al^[2]^ published a systematic review of 113 studies involving 224 endovascular patients treated for ECCA and found a 92.8% procedure success with 93.2% stent-graft patency during the follow-up period. The rates of overall in-hospital mortality, stroke, and cranial nerve injury were respectively 4.1%, 1.8%, and 0.5%, respectively. In our series, the results showed both a higher technique-success ratio and a higher exclusion of the aneurysm sac using endovascular stenting. Such results during the average 24-month follow-up period provided favorable mid-term outcomes in all patients.

Not only do the patients’ conditions determine the choice of ECAA treatment but also aneurysm characteristics such as type, location, and cause should be considered to bring about a favorable outcome. In terms of ECAA type, some researchers found that long-term outcomes after the endovascular procedures were better in the treatment of pseudoaneurysms than true aneurysms.^[20]^ The main reason for this finding is that the causes of the pseudoaneurysms were believed to be self-limited and not likely to reoccur after endovascular therapy, whereas the causes of true aneurysms, such as atherosclerosis, are ongoing and could lead to further aneurysmal degeneration. Another reason is that true aneurysms often have a significant amount of thrombus inside the sac and frequently cause cerebral embolic events during the endovascular procedure compared to pseudoaneurysms.^[18]^ In our series, pseudoaneurysms are the most frequent type of ECAA (8/12, 67%) and the main cause of a pseudoaneurysm was spontaneous dissection and post-traumatic factors. We demonstrated good results in all cases, and all patients were discharged with no neurological dysfunction. However, the limited follow-up duration and numbers of patients do not allow a firm conclusion to be drawn on this issue. Moreover, according to the *Attigah* classification about aneurysm location, high type I and low type V aneurysms might be particularly suitable for an endovascular approach.^[13]^ One reason for this finding is that type II endoleak from the patent ECA, which can be avoided when the aneurysms are far from the bifurcation of the CCA. Another reason for this finding is that these types of aneurysms allow for stent grafts to be chosen because the diameters of the proximal and distal landing zones are similar. In our series of 12 patients, 10 aneurysms were classified as type I or type V. Most aneurysms were excluded through the placement of only 1 stent.

Another thing is the comparisons between covered and bare self-expanding stents. Theoretically, treating aneurysms with covered stents is considered to be more effective for the exclusion of the aneurysm sac, which may lower the rate of endoleak and aneurysm recanalization. According to the meta-analysis of Li et al,^[2]^ a slight decrease in postoperative endoleak, a significant lower rate of reintervention, overall late complications and stent-graft stenosis and a significant increase in thrombosis of the aneurysmal sac can be found in the patients treated with covered stents, which may support the covered stent as a better choice for ECAA. However, the greater profile of covered stent makes its flexibility inferior to that of the bare stent. Moreover, bare stents might easily pass through the tortuous carotid arteries and result in less stent-induced kinking because its superior flexibility is more prone to adjusting to the shape of the vessels.^[21]^ Furthermore, we do not currently have tapered-shape covered stent grafts especially designed for cerebral vasculature use. When the lesions of the aneurysm are adjacent to the bifurcation of the CCA or the diameters of the CCA and ICA exist a considerable gap, treating with regular cylindrical covered stents might increase the risk of postoperative endoleak. In our series, with the exception of 3 patients who were treated with bare stents, all the ECAAs including 3 true aneurysms and 6 pseudoaneurysms were excluded with covered stents. PTFE-covered stents such as Viabahn endoprothesis were most commonly used in our center because of the extreme flexibility and conformability to the vessel configuration and neck movements, which was shown in the report.^[17,22]^ Based on our experience, covered stents are prone to early in-stent thrombosis; therefore, we administered anticoagulation and antiplatelet treatment simultaneously to prevent thrombosis.

Another interesting point regarding our endovascular treatment is that although cerebral protection devices were used only in 1 patient (Patient No. 8) in our series. However, no neurological dysfunction, such as stroke or TIA, occurred in all patients during the procedures. First, compared to carotid artery stenting of atherosclerotic occlusive disease treatment, the widely patent lumen of most ECAAs would theoretically at least imply a lower risk of embolism when stents pass across the lesions during an endovascular intervention.^[23]^ Second, when using covered stents to treat ECAA, a 0.035 inch super stiff guidewire is required as a good support in practice. However, at present, no cerebral protection devices are available that can pass through a 0.035 inch guidewire. These facts supported the rare use of cerebral protection devices in our series.

Though endovascular treatment of ECAA has gained increasing popularity for being a less invasive approach, it is not free from disadvantages and complications. The major post-interventional complications are early thrombosis, restenosis, and occlusion of the stent grafts. Therefore, adequate anticoagulation should be initiated after stent implantations with particular attentions focused on monitoring graft patency. According to a systematic review, the postinterventional restenosis rate is ∼6%.^[2]^ Available evidence on restenosis after carotid artery stenting in ECAA suggests that restenosis appears usually in stents located adjacent to a bend or kinked carotid artery.^[24]^ The only case of recurrent restenosis in our series occurred in a covered stent, which possibly resulted from deploying the stent in the bend of the carotid artery segment. Another limitation of endovascular therapy is that the compression symptoms caused by the aneurysm cannot be eliminated while the mass still exists. Occasionally, the presence of a neck hematoma could lead to aggravation of cranial nerve injuries regardless of the effectiveness of the endovascular procedure, and in some cases, the drainage of the hematoma is required.^[18]^ In our series, 2 patients presented with cranial nerve injuries from aneurysm compression. After successful endovascular stenting, one of them still had the hoarseness similar to the preoperative situation. Whereas another patient's symptom, which presented as choking when drinking, diminished in the follow-up period. It can be assumed that the aneurysm sac would undergo remodeling after the aneurysm exclusion from circulation, but long-term follow-up would be required to address this conclusion. In addition, covered stent was not an option for a kinked ICA with an aneurysm because of the difficulty of endovascular approach and high embolization risk. Nigro et al^[25]^ suggested a hybrid technique with a more favorable outcome, which consisted of surgical shortening of the proximal ICA to adjust the kinking followed by stent placement. This technique was also used in 1 case of our series. We believe such a hybrid technique application in this scenario has potential benefits in the long-term patency of the stent graft.

The primary limitation is the retrospective and nonrandomized nature of the present study in which some inherent selection bias is inevitable. Second, in this study, each patient has different etiology, aneurysm type, and morphologic feature. Additionally, different types of stents, access modes, and postoperative anticoagulation medicine had been used. These heterogeneities in this series could be eliminated through subgroup or confounder-adjusted analysis. Unfortunately, the small sample size as a result of the low incidence of ECAAs makes it difficult to implement any statistical analysis. Therefore, these factors defy any definite conclusion. Another shortcoming is the lack of records of the information about the open surgical and conservative treatments for ECAAs that was mentioned in our article. So we could not get the results regarding the efficacy of 1 intervention compared to another in the long-term follow-up. Moreover, the considerable variation in follow-up times made the long-term results underdetermined. Finally, this was a single-center but not a population-based study with significant treatment heterogeneity.

## 5 Conclusions

Endovascular stenting for ECAAs has promising mid-term results. It should always be an alternative option when treating aneurysms located in the higher position in the internal carotid artery, or when the patient is unfit for open surgery. However, a larger series and a longer follow-up are necessary to further explore the safety and effectiveness of these interventions. Additionally, more researches and development to optimize stent graft designs for endovascular purpose are required to break the present limitations.
